# A normalization model for repeated letters in social media hate speech text based on rules and spelling correction

**DOI:** 10.1371/journal.pone.0299652

**Published:** 2024-03-21

**Authors:** Zainab Mansur, Nazlia Omar, Sabrina Tiun, Eissa M. Alshari

**Affiliations:** 1 Center for AI Technology (CAIT), FTSM, Universiti Kebangsaan Malaysia, UKM, Bangi, Malaysia; 2 Department of Computer Scence, Ibb University, Ibb, Yemen; Kitami Institute of Technology, JAPAN

## Abstract

As social media booms, abusive online practices such as hate speech have unfortunately increased as well. As letters are often repeated in words used to construct social media messages, these types of words should be eliminated or reduced in number to enhance the efficacy of hate speech detection. Although multiple models have attempted to normalize out-of-vocabulary (OOV) words with repeated letters, they often fail to determine whether the in-vocabulary (IV) replacement words are correct or incorrect. Therefore, this study developed an improved model for normalizing OOV words with repeated letters by replacing them with correct in-vocabulary (IV) replacement words. The improved normalization model is an unsupervised method that does not require the use of a special dictionary or annotated data. It combines rule-based patterns of words with repeated letters and the SymSpell spelling correction algorithm to remove repeated letters within the words by multiple rules regarding the position of repeated letters in a word, be it at the beginning, middle, or end of the word and the repetition pattern. Two hate speech datasets were then used to assess performance. The proposed normalization model was able to decrease the percentage of OOV words to 8%. Its F1 score was also 9% and 13% higher than the models proposed by two extant studies. Therefore, the proposed normalization model performed better than the benchmark studies in replacing OOV words with the correct IV replacement and improved the performance of the detection model. As such, suitable rule-based patterns can be combined with spelling correction to develop a text normalization model to correctly replace words with repeated letters, which would, in turn, improve hate speech detection in texts.

## 1. Introduction

The meteoric emergence of social media platforms has piqued research interest in topics such as mining opinion and sentiment analysis. However, the exponential growth of social media has also increased the prevalence of harmful practices, such as abusive language [1], hate speech, and hate-based groups and events [2]. Across the globe, internet extremism and hate are major problems [3], and all social media companies endeavor to delete hateful content before it is posted.

Twitter more commonly uses automated hate speech identification in its texts [4–8]. About 500 million users are registered on Twitter, which is the most dominant microblogging website around the world [9]. As such, the company has increased connectivity among people worldwide and is a convenient public forum for users. However, the language incorporated into Twitter and various social media networks has evolved [10], whereby most users use slang words when writing. The use of slang-style writing has increased the use of out-of-vocabulary (OOV) words, including misspelled words, emoticons, abbreviations, and words with repeated letters [11,12].

Letters are often repeated in written words to emphasize or express feelings. According to [13], the deliberate lengthening of words in microblogs and social media messages is significantly correlated with subjective sentiment. An automatic method was developed to leverage this association and identify domain sentiments and emotional words. [14] agreed that words are lengthened in a sentence to emphasize an expressed opinion. However, if left unaddressed, words with repeated letters decrease the efficacy of natural language processing (NLP) methods [15]. Hate words with repeated letters can appear in different forms in a text. For instance, the letters in a word can be repeated more than once and at various positions. Therefore, learning algorithms need to learn these words differently, as small changes in the input content affect the efficacy of hate speech detection [16,17]. Furthermore, most hate speech detection models are unable to recognize many noisy words in a text due to their lexical discrepancies [18]. Therefore, these noisy words require text normalization to be converted into clearly written texts [19] before proceeding with hate speech detection.

Text normalization has been applied to enhance many NLP tasks for social media texts [9,20,21]. However, in relation to the identification of hate speech on Twitter, the effect of text normalization has not been examined in detail, nor its ability to tackle lexical variants and improve learning performance [22]. Therefore, this present study proposed an improved normalization model for words with repeated letters or OOV words on Twitter. The improved normalization model is an unsupervised method and does not require a special dictionary or annotated data. It combines rule-based patterns of words, repeated letter scenarios, and the SymSpell spelling correction algorithm by [23] to decrease OOV word issues. The goal of this present study was to convert OOV words that occur due to repeated letters to the in-vocabulary (IV) words that are found in the dictionary. The two most significant contributions of this present study include a pattern-based detection of OOV words and improving the method of normalizing OOV words into correct IV words.

The remainder of this research is organized as follows: The Related Works section contains related studies on the normalization of OOV words. The Research Methods section covers the analysis of the letter repetition patterns of OOV words. The Experimental Evaluation and Results section describes the improved pattern-based repeated letter normalization model and spell checker. The Discussion section presents a discussion of the results. The Contributions section elucidates the research contributions. The Conclusion and Future Work section provides suggestions for future studies.

## 2. Related works

Text normalization on Twitter requires more nuance than short message service (SMS) as Twitter contains richer lexical variants and is a noisier data source [24]. Furthermore, as the language used with social media is continually evolving, it causes new errors in word patterns. Every generation has introduced different writing styles, so every style requires different normalization methods [25]. Therefore, social media texts like tweets must be pre-processed or normalized to remove all noise before they can be appropriately structured. This is because non-normalized tweets may be rife with issues, such as incorrect grammar, freestyle words, spelling errors, and abbreviations [20]. This has motivated the NLP community to shift towards text normalization, which involves converting OOV words into IV words [19,26–30].

However, as users intentionally obscure words to escape detection, it significantly inhibits the success of hate speech detection [18]. An example of this would be the extra-long words commonly used in abusive messages [31]. Although text normalization strategies have improved several NLP tasks for social media texts, the benefits of these strategies in the context of hate speech detection on Twitter have not been explicitly discussed [22]. Social media users repeat characters to convey a message or emphasize a point [13]. [13] used several steps to detect lengthy OOV words and match them with a standard IV form of the words in question. The repeated letters were first replaced with a single instance before combining other words that share the same form. Sets that did not contain a repetition of three letters were then removed before the most common form within the group was chosen and adopted as the standard IV form of the word in question.

Meanwhile, multiple other studies [32–37] utilized the regular expression method to tackle OOV words. In the regular expression method, a pattern such as the (r” (\w)\1+”) format is set up to delete words that contain a certain number of repeated letters. However, [33] used the regular expression method to compile the patterns of the repeated letters and then used the replace () method to generate a more accurate version of each word in the pattern. Their removal of repeated characters is questionable [38]. [33] used the regular expression method to replace letters that appeared three consecutive times with two of same letter. Similarly, [34] the regular expression method to delete repeated letters based on a match pattern of Malay tweets. Meanwhile, [36] used a regular expression function to remove repeated letters until only two letters remained.

Likewise, [14] used the regular expression method to pattern-match words ending with three or more repeated letters. [35] developed a system to remove repeated letters from a word one at a time. The system runs a WordNet lookup each time a letter is deleted. Whenever WordNet finds a word, it stops eliminating repeated letters. [37] only replaced words with more than three repeated letters and only if the word appeared in the text more than three times, whereas [31] replaced words with two repeated letters and those that appeared more than twice.

[12,38,39] recently yielded promising results by using the regular expression method followed by a spell-check algorithm when the suggested IV replacement word was incorrect. More specifically, [12] reduced repeated letters to two letters while [39] reduced them to one letter. Both studies used a spell-checking algorithm after using the regular expression method. However, neither method had determined the correctness of the suggested IV replacement words.

Multiple studies have successfully developed rule-based methods for many languages and tasks, which yielded impressive results [21,25,34,40–46]. However, new rules have to be developed to detect new error patterns that emerge due to changes in social media language usage [25]. Furthermore, little effort has been made to establish rules for normalizing words with repeated letters on Twitter based on the pattern scenarios of repeated letters and to distinguish between correct and incorrect IV replacement words.

The SymSpell algorithm has recently been used to address spelling correction issues [47–49]. The algorithm efficiently checks and provides suggestions [50]. It also uses a new method of dictionary searching, which significantly increases performance and language independence [49]. The SymSpell algorithm also checks whether an OOV word is misspelled and uses the Levenshtein distance to suggest the closest correctly spelled IV word.

Therefore, a normalization method that integrates rule-based patterns for words, repeated letter scenarios, and the SymSpell spelling correction algorithm may provide a better solution for OOV words and enhance the model used to detect hate speech. This would, in turn, overcome the limitations of extant methods and enrich the learning process of detecting hate speech text by decreasing OOV words.

## 3. Research method

**Fig 1:**
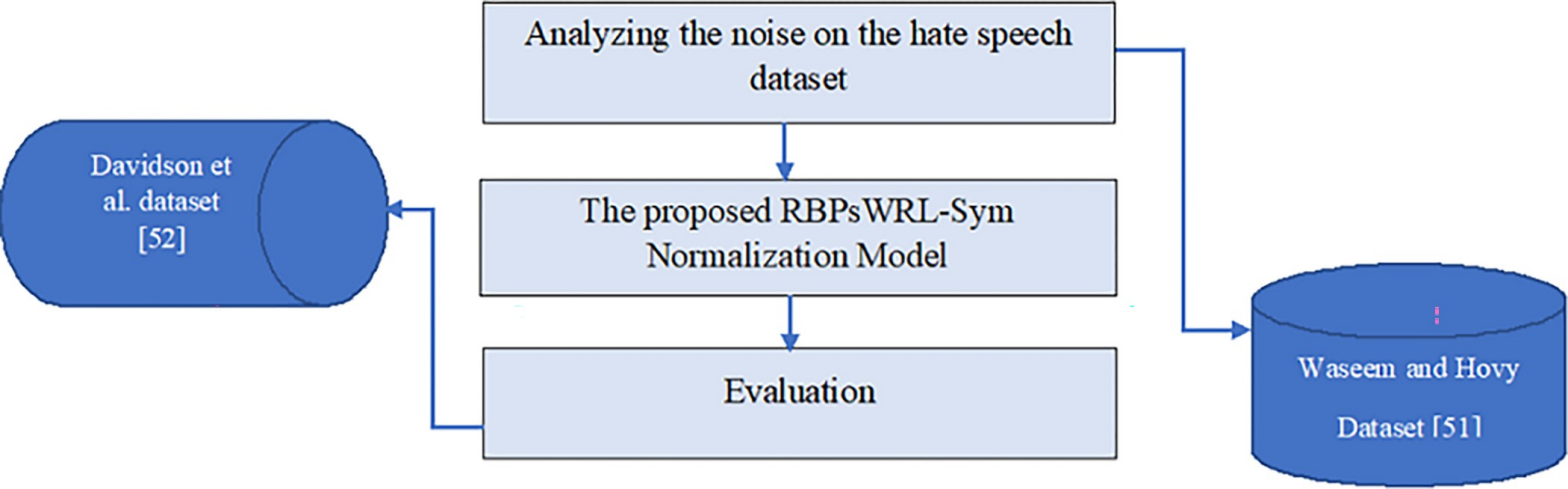
Methodology of the proposed normalization model.

Fig 1 depicts the proposed method of normalizing repeated letters of OOV words. The three main stages include: (i) identifying the letter repetition pattern of OOV words based on a training dataset of hate speech by [51], (ii) proposing a rule-based pattern for words and SymSpell correction (RBPsWRL-Sym) normalization model based on pattern and spelling, and (iii) evaluating the effectiveness of the projected normalization model using a dataset by [52]. The following subsections provide detailed explanations of each of the stages.

### 3.1 Pattern identification of words with repeated letters

Previously published works by [53–55] have raised awareness that patterns can be identified by analyzing data.

The pattern of repeated letters in words was analyzed from the dataset [51].

The dataset by [51] is a hate speech tweet dataset written in English which consists of 16914 tweets labeled racist, sexist, or neither. The classes in this dataset contained 3,383 sexist tweets, 1972 racist tweets, and 11559 tweets that were neither sexist nor racist. The data set is freely available as tweet IDs and labels on Github. Two types of analyses were conducted on the dataset by [51]: (1) to identify unknown words with repeated letters and (2) to identify the letter repetition pattern of these unknown words. To perform both these analyses, a spelling checker tool; namely Pyspellchecker [56] was used to check the spelling of the words from the dataset.

**Fig 2:**
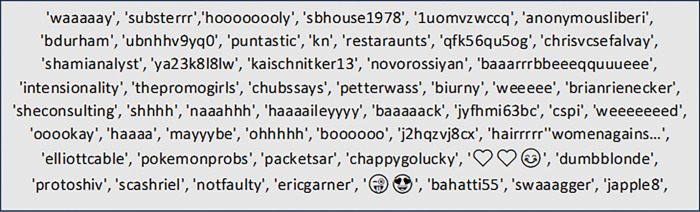
Sample of unknown words from the dataset by [51].

The Pyspellchecker supports Python programming language and is based on the method proposed by [57]. Pyspellchecker is multi-language compatible and uses the Levenshtein distance to obtain probabilities within an edit distance of two from the entered word. Therefore, Pyspellchecker was used to determine if a word was known or unknown. Table 1 shows the data for all the words. This includes words that are both known and unknown. A total of 11417 (47%) of the dataset by [51] contained unknown words. As seen in Fig 2, words with repeated letters frequently appear as unknown words.

**Table 1:** Known and unknown words found in the dataset on hate speech by [51].

Hate speech dataset by [51] Categories Word count	
Number of total words	24384
Number of known words	12749
Number of unknown words	11417

Based on the manual analysis of the unknown words, words with repeated letters appeared most frequently. As a preliminary step, the patterns of several words with repeated letters were examined (Table 2). As multiple letters are sometimes repeated at various positions in a word (Table 2), the current normalization strategy needs to be changed. As seen in Table 2, an analysis of these patterns revealed which words require special processing. Therefore, developing an algorithm based on these patterns will result in more successful normalization outcomes. Towards that end, a model containing several algorithms, i.e., the RBPsWRL-Sym model was developed based on the analysis (Table 2). The designed model could efficiently handle words with a single repeated letter, such as ’aaaand’, along with multiple repeated letters, such as ’shhhhhiiiiit’.

**Table 2:** Patterns of letter repetitions and positions in words.

Pattern	Repeating scenario	Example of words with repeated letters	Correct word form
Pattern 1	At the beginning of the word scenario	wwweek, aaaand	week, and
Pattern 2	The single case middle word scenario	waaaay, baaaaack	way, back
Pattern3	The double case middle word scenario	cheeeeezzzzzy	cheezy
Pattern 4	The end scenario	scrollllllllll, wayyyyy	scroll, way
Pattern 5	An abbreviation with repeated ending letters	lollllllll, grrrr	Laughing out loud, great
Pattern 6	Combination of patterns # 1,2,3,4	aaannndddd	and
Pattern 7	The beginning and the ending of the word scenario	nnnnnoooooooooo, ssoooo	No, so
Pattern 8	The beginning and middle scenario	mmmmaaaaan	man
Pattern 9	The middle and the end of the word scenario	foooooddddd	food

### 3.2 The proposed RBPsWRL-Sym normalization model

**Fig 3:**
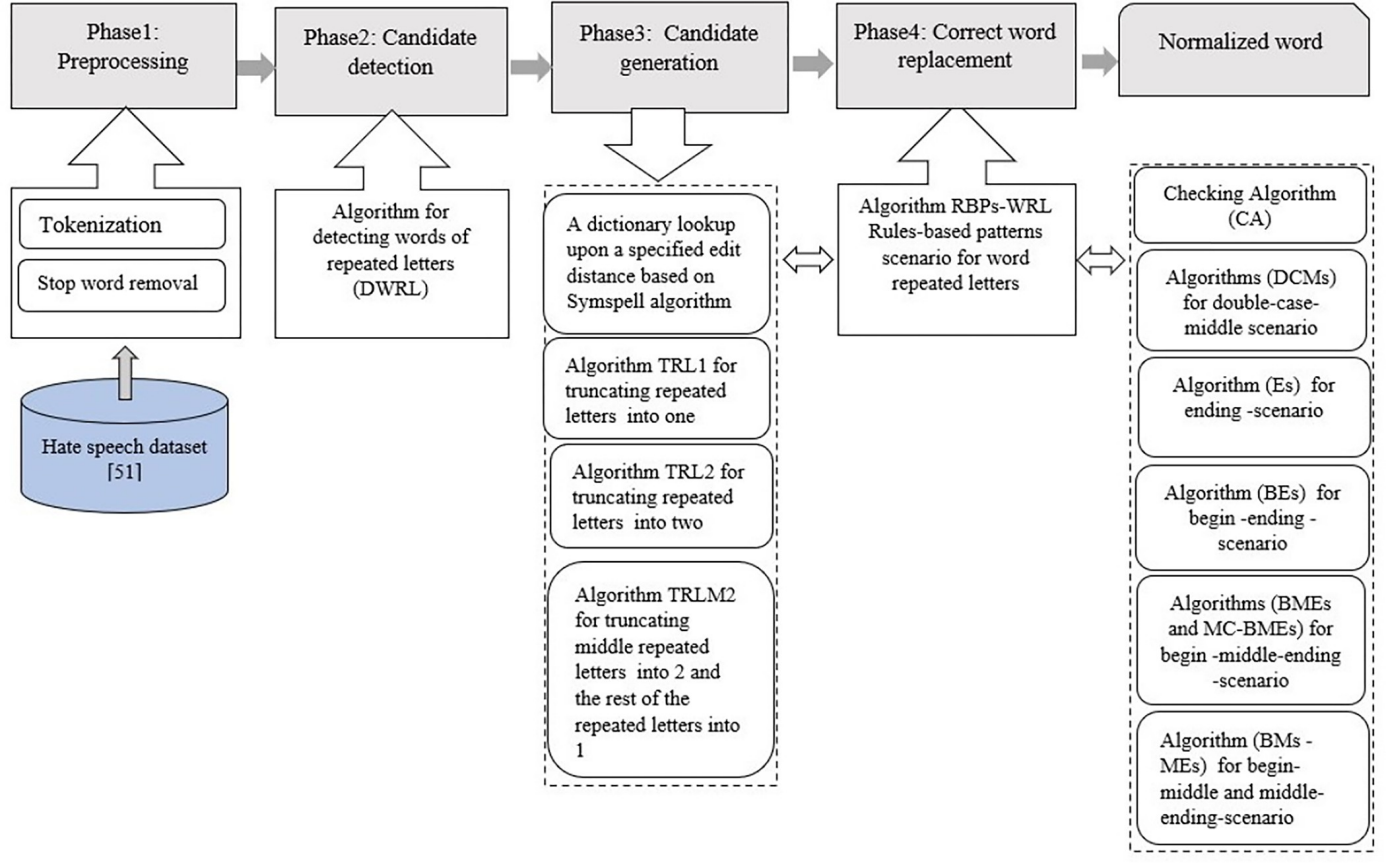
Diagram of the proposed RBPsWRL-Sym normalization model.

The proposed normalization model, i.e., the RBPsWRL-Sym model, is based on the rule-based pattern scenario (RBPs) for words with repeated letters (WRL) and the SymSpell spelling correction algorithm (Sym). The RBPsWRL-Sym model was designed to suggest the best IV replacement words for OOV words with repeated letters. Fig 3 depicts the overall RBPsWRL-Sym model. It consists of four phases: (i)Text pre-processing, (ii) candidate detection, (iii) candidate generation, and OOV word replacement with correct (IV) word. The subsequent subsections describe each phase in greater detail.

#### 3.2.1 Phase 1: Text pre-processing

The first phase of the RBPsWRL-Sym normalization model is text pre-processing, which involves tokenization and stop-word elimination. Removal of stop words is the process of removing words that are commonly not informative. This includes articles, prepositions, pronouns, and conjunctions. English stop words include “a,” “an,” “the,” “so,” and “what.” Tokenization involves splitting a text into groups of words called tokens. These tokens must be normalized to their original format. As such, tokenized words are passed through the proposed normalization scheme on a word-by-word basis. This is described in greater detail in the succeeding subsections.

#### 3.2.2 Phase 2: Candidate detection

It is integral that OOV words are identified in the first step of the normalization process. Therefore, a detection algorithm is used to check every word in a tweet to determine if it is an OOV word that contains repeated letters or not.

**Fig 4:**
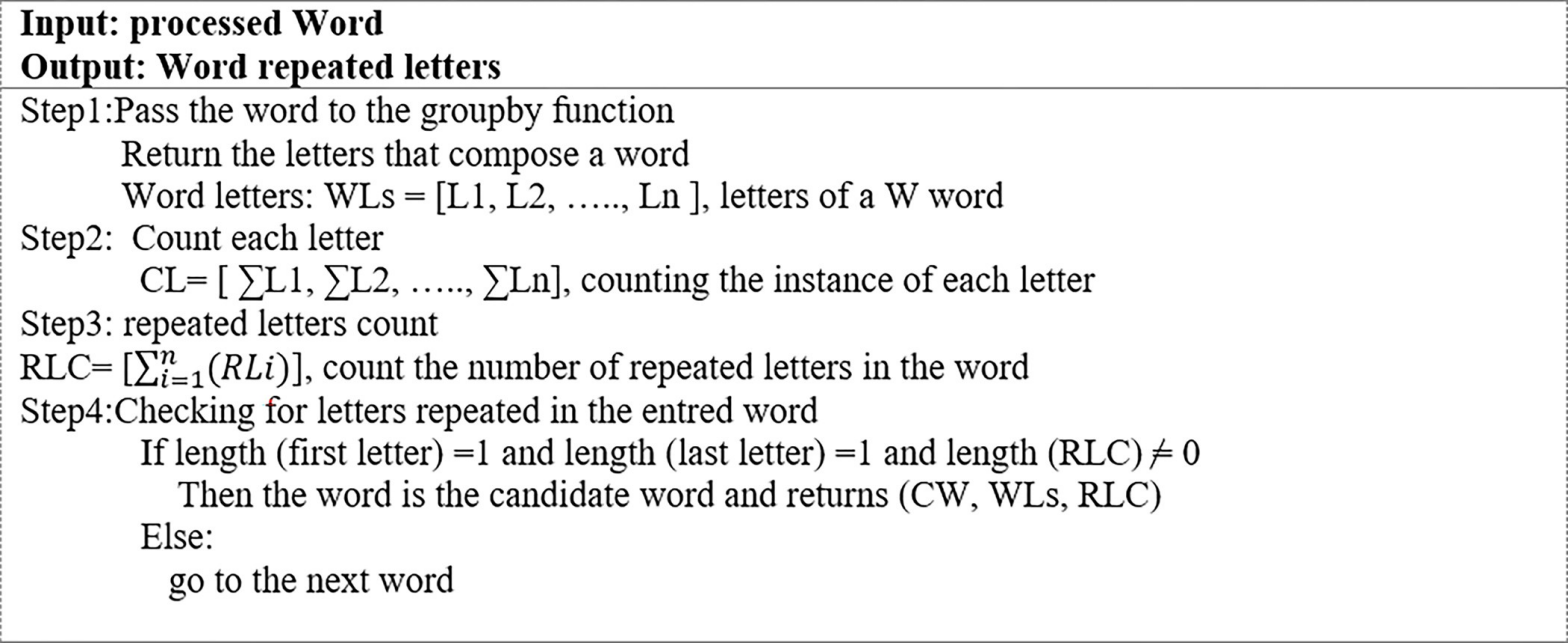
The CDWRL algorithm.

Fig 4 shows the algorithm for the proposed candidate detection of words with repeated letters (CDWRL). In Step1, every input word is sent to the GroupBy function to split the word into its constituent letters. The GroupBy function divides data into separate groups to facilitate analysis and rule generation. The function returns an output list of word letters (WLs) and the total number of each letter in a word. In Step2, the number of times each letter appears in a word is determined and stored in the letters count (LC) variable.

**Fig 5:**
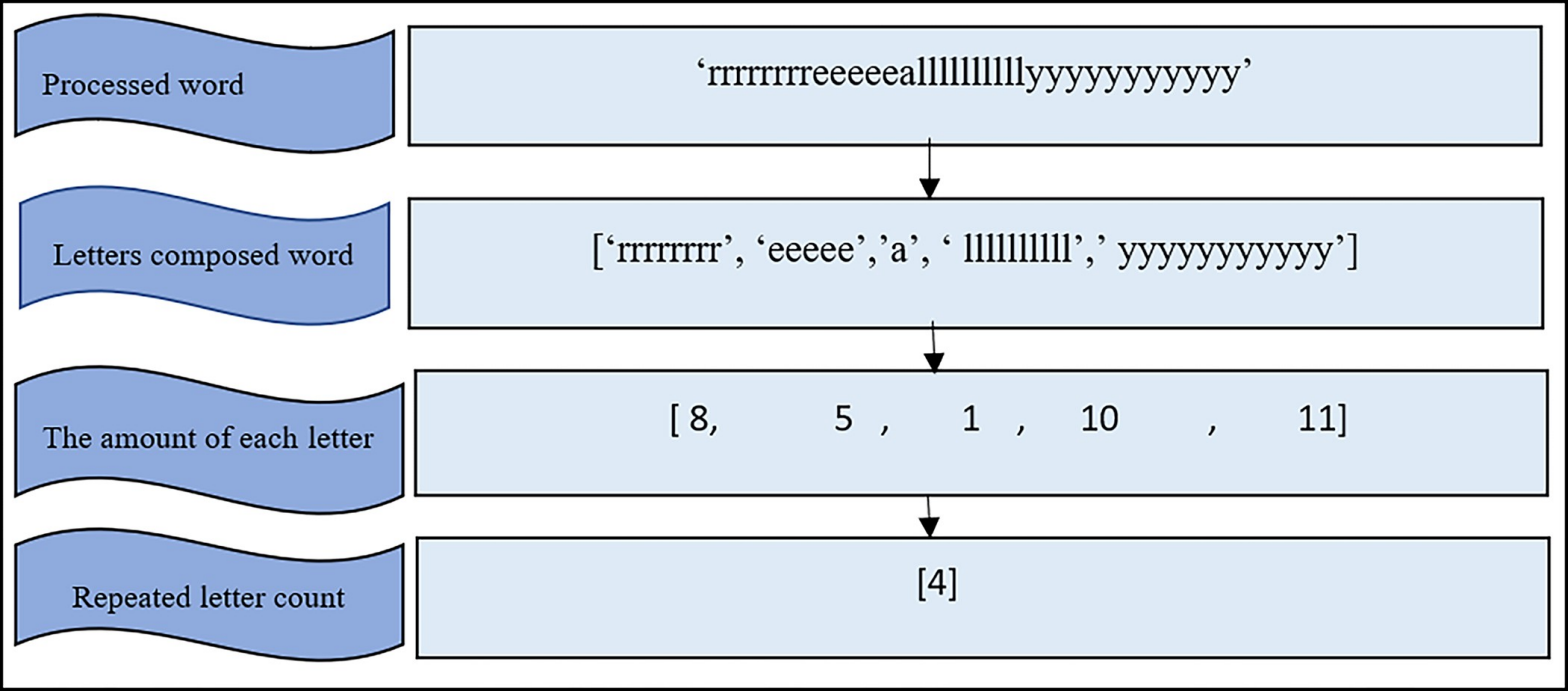
Example of outputs generated in Step1 to 3 of the CDWRL algorithm.

In Step3, the number of times each letter is repeated in a word is determined and stored in the repeated letters count (RLC) variable. Lastly, in Step 4, the RLC variable is used to determine if a word is an OOV word or not. If the RLC = 0, the word is not a candidate; otherwise, it is. Fig 5 depicts the output process of the algorithm as well as examples of steps 1 to 3.

#### 3.2.3 Phase 3: Candidate generation

In this phase, IV words that may be the correct form of OOV words with repeated letters are generated. These OOV words are corrected at the word level only, excluding the context in which they are used. Users often lengthen a word by repeating letters to emphasize meaning without significantly changing the form of the word. At the candidate generation phase, candidate IV words are generated for an input OOV word using four algorithms namely: (i) SymSpell spelling correction, (ii) truncation of repeated letters to one (TRL1), (iii) truncation of repeated letters to two (TRL2), and (iv) truncation of repeated letters in the middle of a word to two (TRLM2). Instead of using the regular expression method to remove repeated letters, these three algorithms namely TRL1, TRL2, and TRLM2 are developed to manipulate the letters of a word. The generated IV candidate words are later used to determine the best replacement for the OOV word in question.

- **SymsSpell spelling correction algorithm**

According to the Levenshtein distance, the SymSpell spelling correction algorithm of [23] looks up suggestions of the closest correctly spelled IV word. This is an enhanced version of the Norvig spelling correction method [57], as it generates all the IV words using only a delete editing operation at a distance of N to the searched OOV word and looks for it in a word frequency dictionary. However, the Symspellpy library must be installed in the proposed normalization method before executing the SymSpell algorithm.

**b. Algorithm for truncating repeated letters to one (TRL1)**

**Fig 6:**
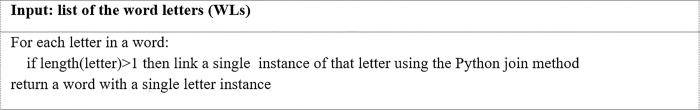
The TRL1 algorithm.

The purpose of this algorithm is to remove all the letters repeated in a word so that each letter appears only once. Fig 6 offers the TRL1 algorithm for the process of truncating repeated letters to one instance.

**c. Algorithm for truncating repeated letters to two (TRL2)**

**Fig 7:**
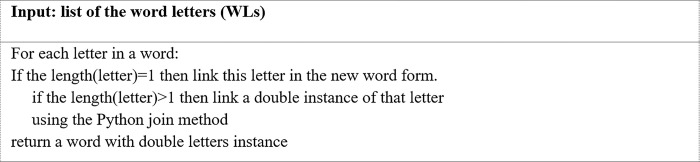
The TRL2 algorithm.

The purpose of this algorithm was to remove all the letters repeated in a word so that each letter appears twice. Fig 7 illustrates the TRL2 algorithm for the process of truncating repeated letters into two instances.

**d. Algorithm for truncating repeated letters in the middle of a word to two (TRLM2)**

**Fig 8:**
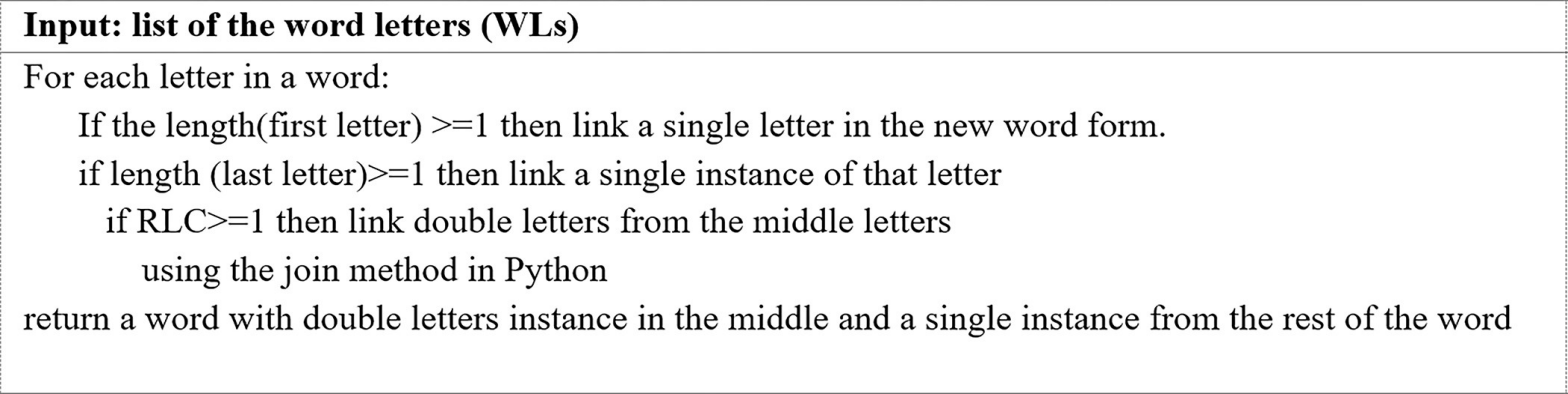
The TRLM2 algorithm.

Some words require special treatment, such as decreasing the number of letters at all positions in a word to one instance and from the middle of a word to two. The TRLM2 algorithm was designed for this purpose. Fig 8. illustrates the TRLM2 algorithm for the process of truncating repeated letters in the middle of a word to two instances.

**Fig 9:**
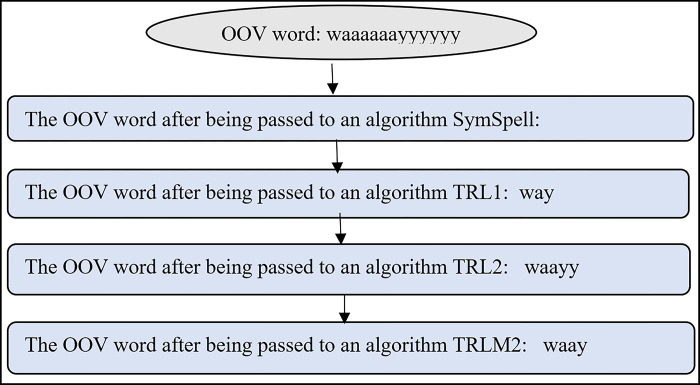
Example of correct word candidates generated for the OOV word ‘waaaaaayyyyyy’.

**Fig 10:**
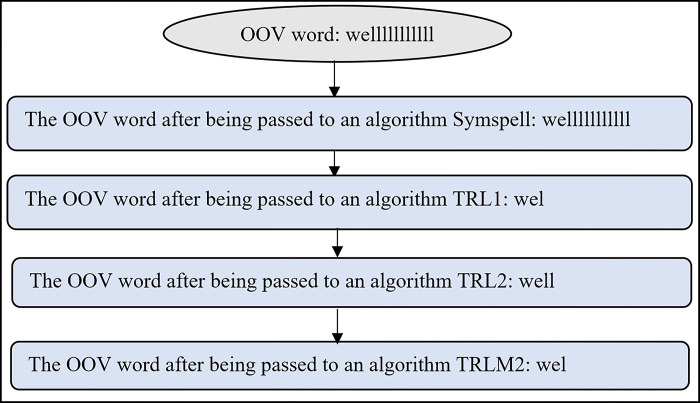
Example of correct word candidates generated for the OOV word ‘welllllllllll’.

All the above algorithms were designed to generate potentially correct word candidates, which will be examined later in the word replacement process. Figs 9 and 10 illustrate two examples of outputs from the above algorithms (Figs 6–8). As seen in Fig 9, three candidates, ‘way,”waayyy’ and ‘way’; were proposed as replacement words for the OOV word ‘waaaaaayyyyy,’ while two candidates; ‘well’ and ‘well’; were submitted as replacement words for the OOV word ‘welllllll’ (Fig 10). Only one of these correct word candidates will be selected to replace the OOV word.

#### 3.2.4 Phase 4: Replacement with correct in-vocabulary (IV) word

**Fig 11:**
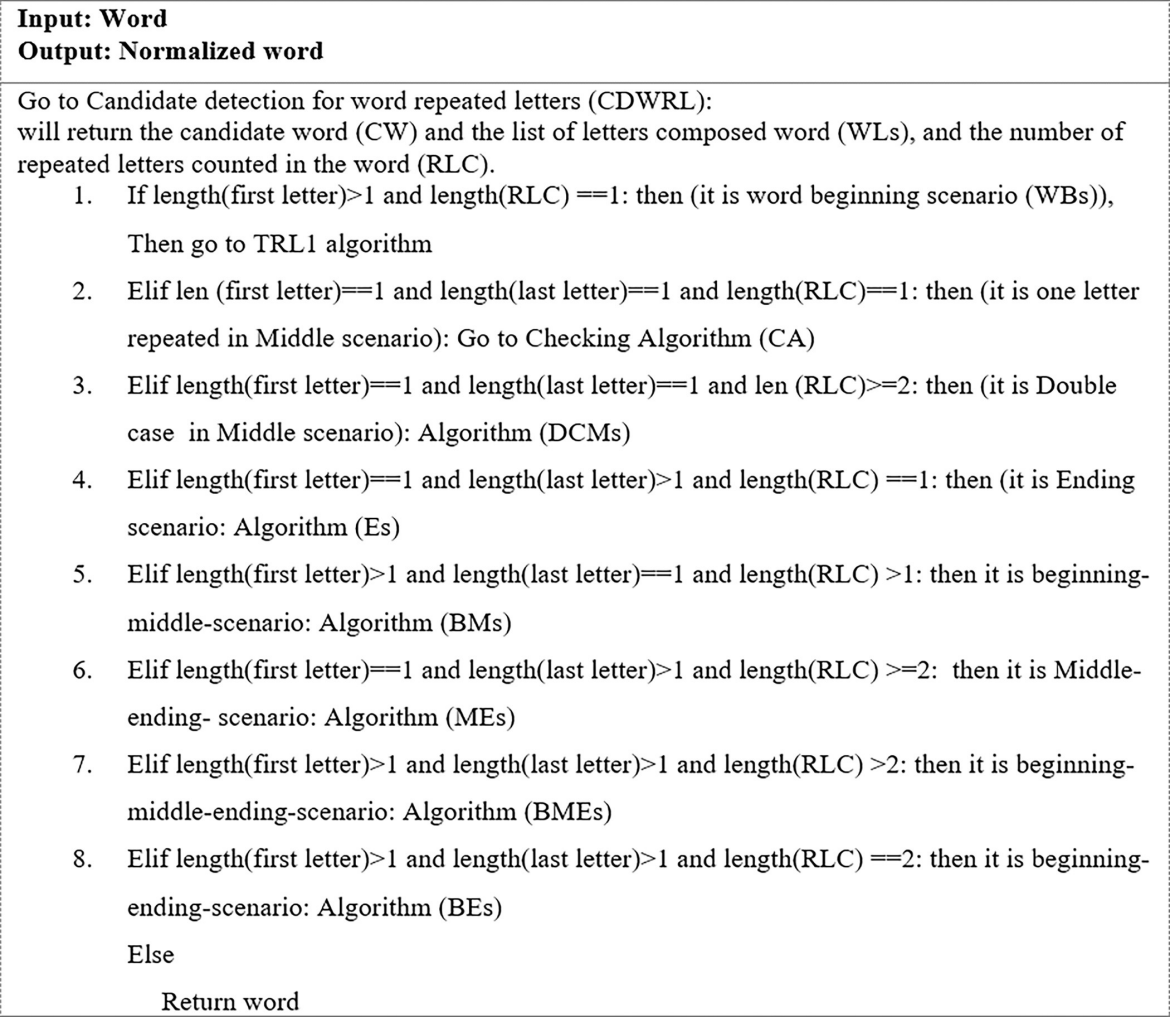
The RBPsWRL-Sym algorithm.

The last and most critical aspect of the normalization process is replacing OOV words with correct IV words. The OOV word replacement method relies on the word repeated letters pattern scenario. In this phase, the letter repetition patterns are the patterns developed as described in Pattern identification of words with repeated letters section (Table 2). Existing normalization methods require a modification in cases where words contain more than one repeated letter. However, users often repeat letters at different positions in a word. As such, some words must be normalized differently. Therefore, an algorithm that is based on these patterns was developed as it is more likely to produce successful results. Fig 11 illustrates the RBPsWRL-Sym algorithm for the proposed normalization scheme. An OOV word was inputted into this algorithm, and the output was an IV replacement word. The OOV word was then subjected to eight pattern-matching rules (Table 2) to determine the best IV replacement word.

In Pattern 1, one letter at the beginning of a word had been repeated. The TRL1 algorithm is utilized to truncate the letters to one instance. It is uncommon for letters to be repeated more than once in English [39]. It is even rarer for letters to be repeated at the beginning of an English word.

**Fig 12:**
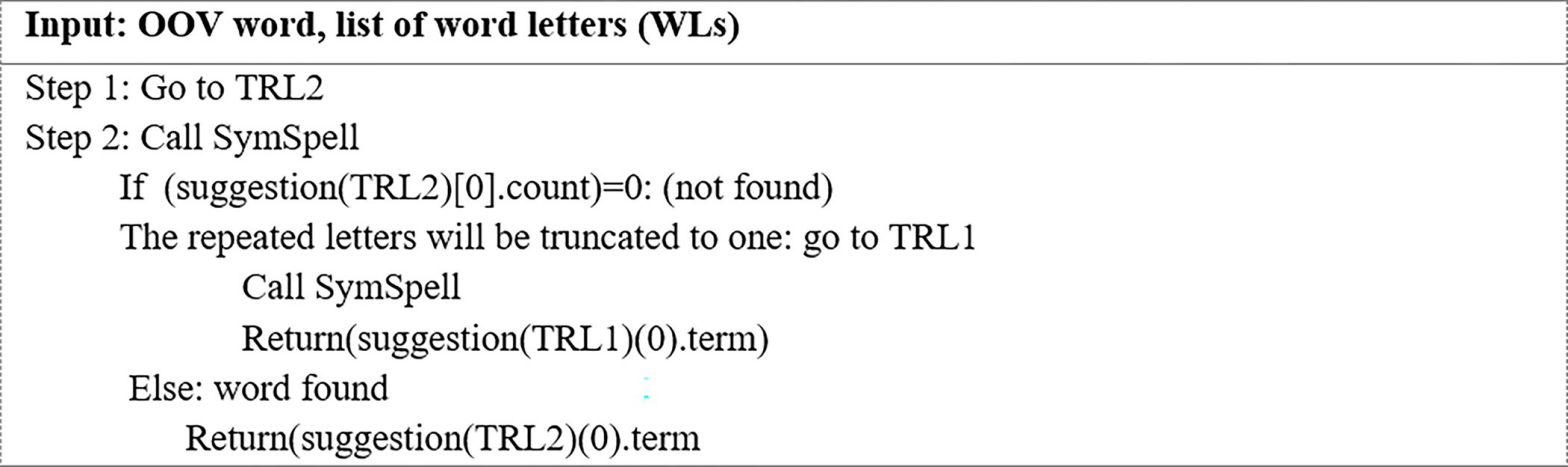
Checking Algorithm (CA).

In Pattern 2, one letter in the middle of a word had been repeated. This algorithm is utilized when the length of the first and last letters = 1 and the RLC = 1. The word is then sent to the checking algorithm (CA) to be processed. Fig 12 depicts the mechanism of the CA algorithm. The OOV word and its constituent letters are inputted into the CA. The CA then truncates all the repeated letters to two letters each before it is sent to the SymSpell algorithm to be checked. The SymSpell algorithm screens the looked-up suggestions for the OOV word and uses the Levenshtein distance to obtain the closest correctly spelled IV word. It then provides an individual search result with the smallest edit distance and highest frequency of word. More specifically, it suggests an IV word only if there is one to suggest. Otherwise, the repeated letters in the OOV word are truncated to a single letter and passed through the provided looked-up suggestions. If the SymSpell algorithm can suggest an IV word, it replaces the input OOV word. Otherwise, no changes are made.

**Fig 13:**
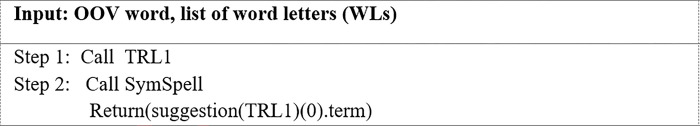
The DCMs algorithm.

In Pattern 3, the input OOV word contains more than one repeated letter in the middle. This algorithm is utilized when the length of the first and the last letters each; = 1 and the RLC is at least two. If the input OOV word matches the pattern case, it is sent to the double-case in the middle scenario (DCMs) as in Fig 13. Unlike the one letter repeated in the middle scenario, the DCMs require a different perspective.

In the DCMs algorithm, the repeated letters in the word are reduced to a single letter. The newly processed OOV word is passed to the dictionary look-up method for further confirmation. It has been observed that users rarely make substantial changes to a word in this pattern.

**Fig 14:**
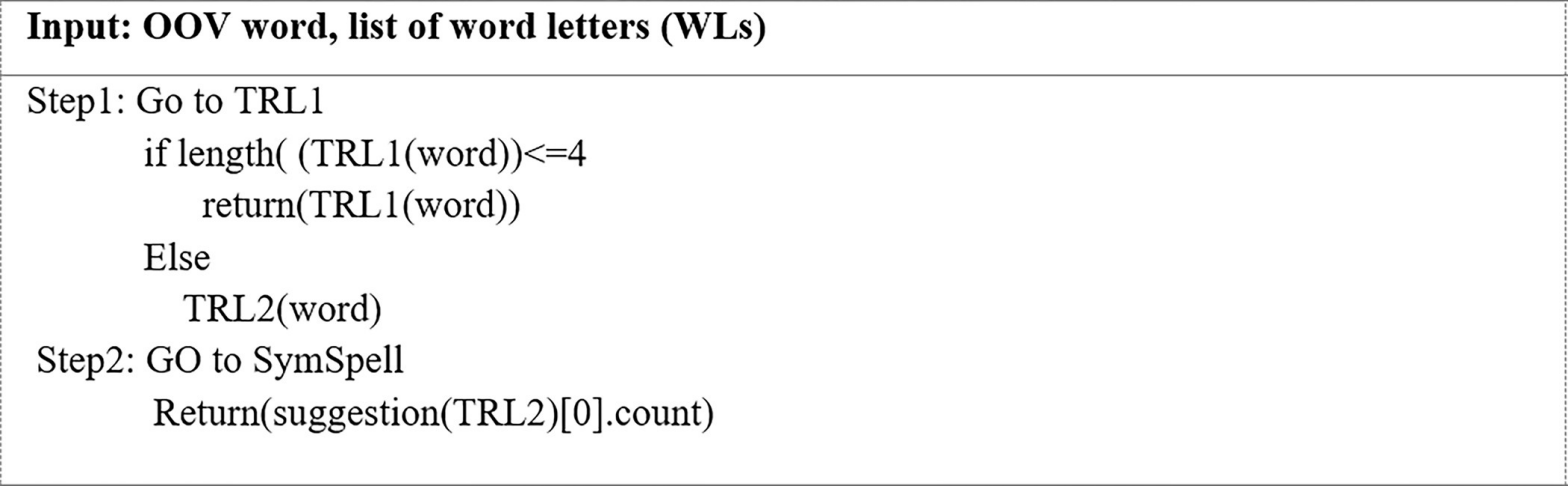
The Es algorithm.

In Pattern 4, the length of the last letter > 1. This algorithm is utilized when the length of the first letter = 1 and the RLC = 1. Several English words contain repeated letters at the end; however, they do not exceed two repetitions. The end scenario (Es) algorithm was developed to handle the case-matching pattern as in Fig 14. This algorithm has different procedures in that the repeated letters are first truncated to one. The length of the truncated word is then checked to determine if it is < 4. An analysis of abbreviations that contain repeated letters at the end indicated that the length of these words is <4. Therefore, if this condition is met, the truncated word replaces the OOV word. Otherwise, the repeated letters of the OOV word that meet this pattern case are truncated to two before it is sent to the dictionary look-up method for further verification, and a replacement will occur.

**Fig 15:**
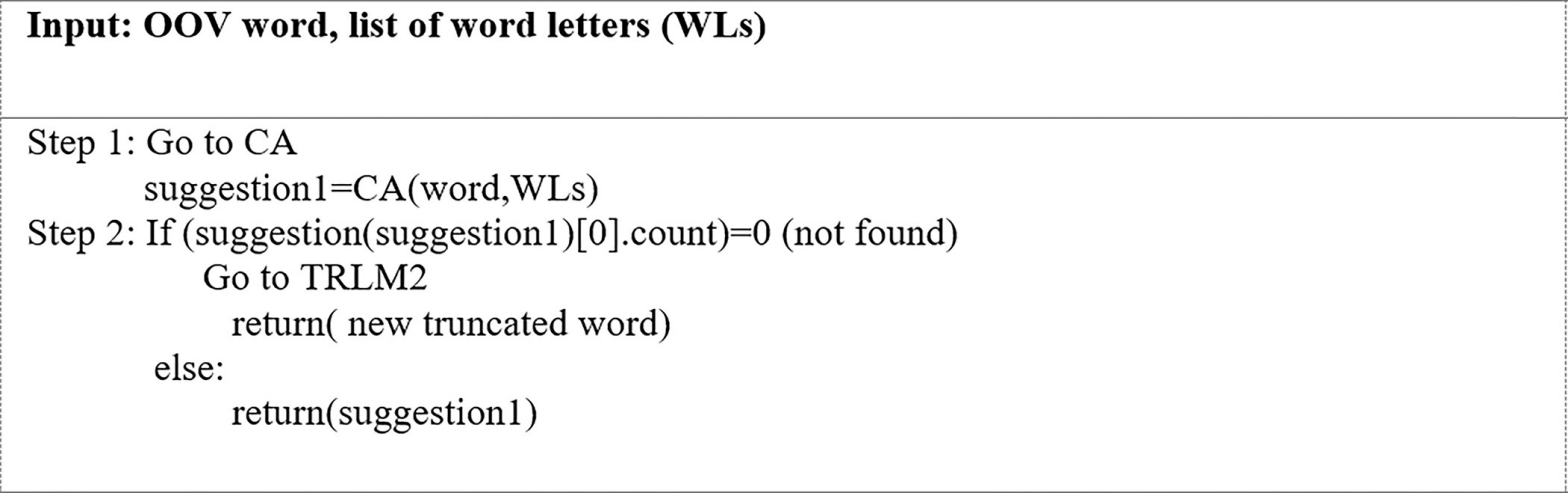
The BMs-MEs algorithm.

In Pattern 5, the input OOV word contains repeated letters at the beginning and the middle positions. This algorithm is utilized when the length of the first letter > 1, the length of the last letter = 1, and the RLC>1. Fig 15 provides the BMs-MEs algorithm designed to manage this pattern and the subsequent pattern. To avoid incorrect word substitutions, the different processes used in this algorithm were based on an analysis of repeated letters and their position in words. After the word and a list of its constituent letters are inputted into the BMS-MEs algorithm, the word is sent to the CA. The CA then returns a word that will be examined again by the look-up algorithm. The word is replaced when a valid word is found. Otherwise, the input OOV word will be sent to the TRLM2 algorithm, and the resulting word will be used as the replacement word.

In Pattern 6, the input OOV word contains > 1 repeated letter at the middle and end positions. This algorithm is utilized when the length of the first letter = 1, the length of the last letter is > 1, and the RLC is > 2. The BMs-MEs algorithm in Fig 15 was developed by analyzing and examining this pattern. Therefore, it can also be used to handle this pattern.

**Fig 16:**
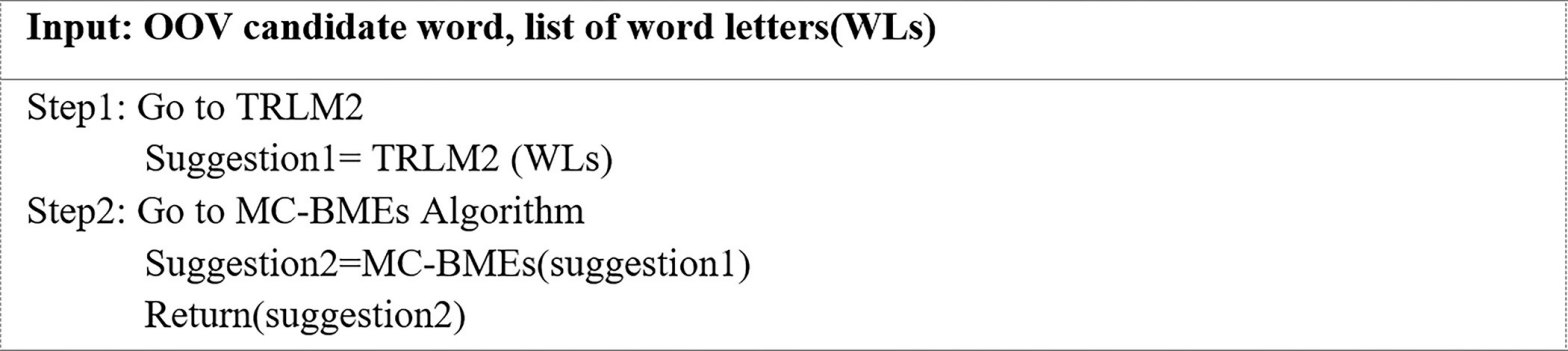
The BMEs algorithm.

Pattern 7 is more complex as letters are simultaneously repeated at three different positions in a word: the beginning, middle, and end. This algorithm is utilized when the length of the first and last letters > 1 and the RLC> 2. Fig 16 offers the beginning-middle-end -scenario (BMEs) algorithm that was designed to manage this pattern. It includes various procedures, such as an algorithm that is based on an analysis of repeated letters and their positions in words to prevent incorrect IV-word choices.

**Fig 17:**
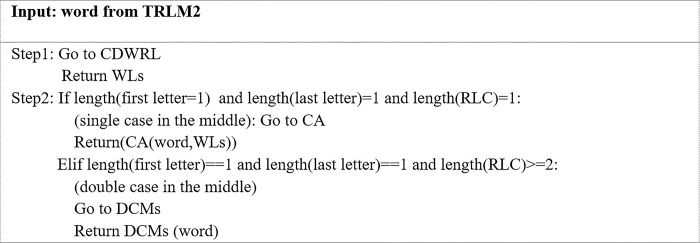
MC-BMEs algorithm.

The inputted OOV word is first sent to the TRLM2 algorithm. The output word is then put through an algorithm specifically developed to handle cases of letter repetition in the middle of words that contain letter repetitions in the beginning, middle, and end (MC-BMEs) to yield a replacement word as in Fig 17. The MC-BMEs algorithm was designed to deal with letter repetitions in the middle of a word that contains letter repetitions in the beginning, middle-, and end, such as ‘cccccaaaaannnnn’. When the OOV word ‘cccccaaaaannnnn’ was inputted in the TRLM2 algorithm, it removed letters from each position and maintained two letters in the middle to generate ‘caann’. This word was then put in the MC-BMs algorithm, and the returned word was sent to the CDWRL algorithm, which detects the OOV candidate word and provides a list of letters in the word. At this point, if the word contains a single letter repetition in the middle position, it is sent to the CA algorithm. If it contains more than one letter repetition in the middle position, it is sent to the DCMs algorithm.

**Fig 18:**
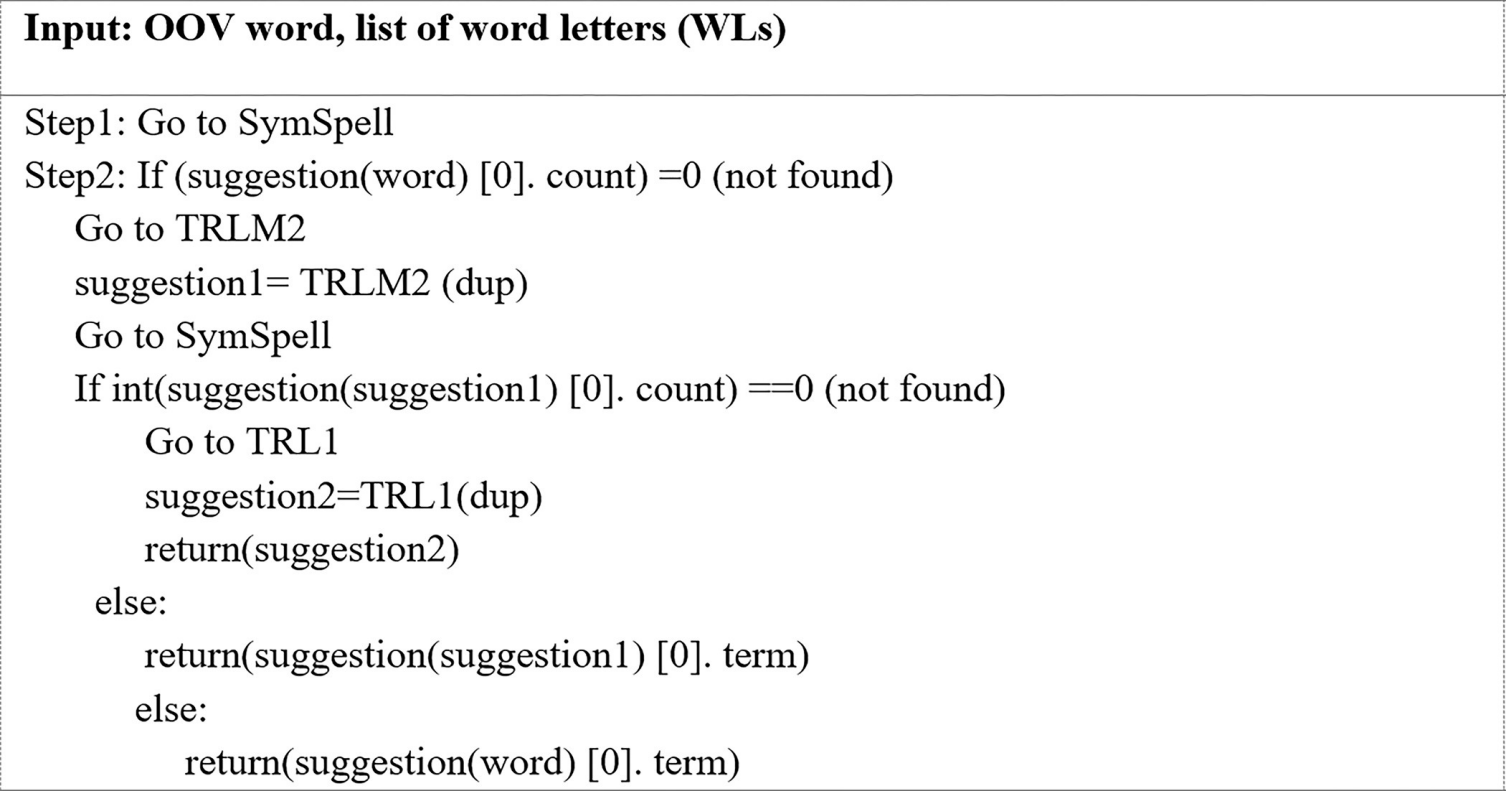
The BEs algorithm.

In Pattern 8, letters are repeated at the beginning and end of words. This algorithm is utilized when the first and last letters are > 1 and two letters are repeated in the word when RLC = 2. The (BEs) algorithm in Fig 18 was designed to manage an instance with a similar pattern. It includes various procedures such as an algorithm that is based on an analysis of repeated letters and their positions in words to prevent incorrect IV-word choices. The input for this algorithm is the OOV word and a list of its constituent letters. The OOV word is first sent to the dictionary look-up method for verification to yield a word with the highest frequency and the lowest distance; normalization is not required if there is a match. Otherwise, the OOV word is sent to the TRLM2 algorithm. The returned word is rechecked for the closest correctly spelled IV-word and then replaced. Otherwise, the returned word is sent to the TRL1 algorithm to truncate all the repeated letters to one letter each before the word is substituted.

The following is an explanation of how the RBPsWRL-Sym normalization model normalized OOV words with repeated letters. For example, letter repetitions in the middle of a word are truncated to two if there is only one repetition. Table 3 provides an example of avoiding the wrong word return from the dictionary look-up method when the letter repeated in the middle is truncated to one. When letters are repeated at the end, they should be truncated to two first. This prevents the wrong word from being returned by the dictionary look-up method by truncating the repeated letter to one (Table 3). When letters are repeated in the beginning, middle, and end of a word, it is significant to avoid having the wrong word returned from the dictionary lookup method when all the repeating letters are truncated to two. Therefore, only letters repeated in the middle will be truncated to two, while the other repeated letters will be truncated to one (Table 4).

**Table 3:** Example of normalizing the word ‘foooooooooood’ and ‘welllllllllllll’ using the proposed method and four other normalization methods.

Word	U.S Standard English dictionary	Regular expression	Regular expression + SymSpell spelling correction	Khan and Lee [39]	Pyspellchecker tool [56]	Our method
foooooooooood	Not Found	fod	for	for	foooooooooood	food
welllllllllllll	Not Found	wel	we	we	welllllllllllll	well

**Table 4:** Example of normalizing the word ‘aaaaaaannnnnndddd’ using the proposed method and four other normalization methods.

Method	U.S. Standard English dictionary	Regular expression	Regular expression + SymSpell spelling correction	Sosamphan et al. [12]	Pyspellchecker tool [56]	Our method
Output word	Not Found	aanndd	banned	manned	aaaaaaannnnnndddd	**and**

## 4. Experimental evaluation and results

Experiments were conducted using different datasets to examine the effectiveness of the projected normalization method. The first experiment was conducted using the dataset by [51] to assess the ability of the normalization algorithm to reduce OOV. The second assessment was carried out using the dataset by [52] to assess the effectiveness of the projected normalization scheme compared to other normalization methods. The dataset in [52] was collected from Twitter and divided into three classes: hate speech, offensive language, and neither. It was manually coded by CrowdFlower (CF) workers, and the intercoder-agreement score reported by CF is 92%, which results in a sample of 24802 labeled tweets.

### 4.1 Experiment 1: The effect of the RBPsWRL-Sym normalization model on the reduction of OOV words

**Fig 19:**
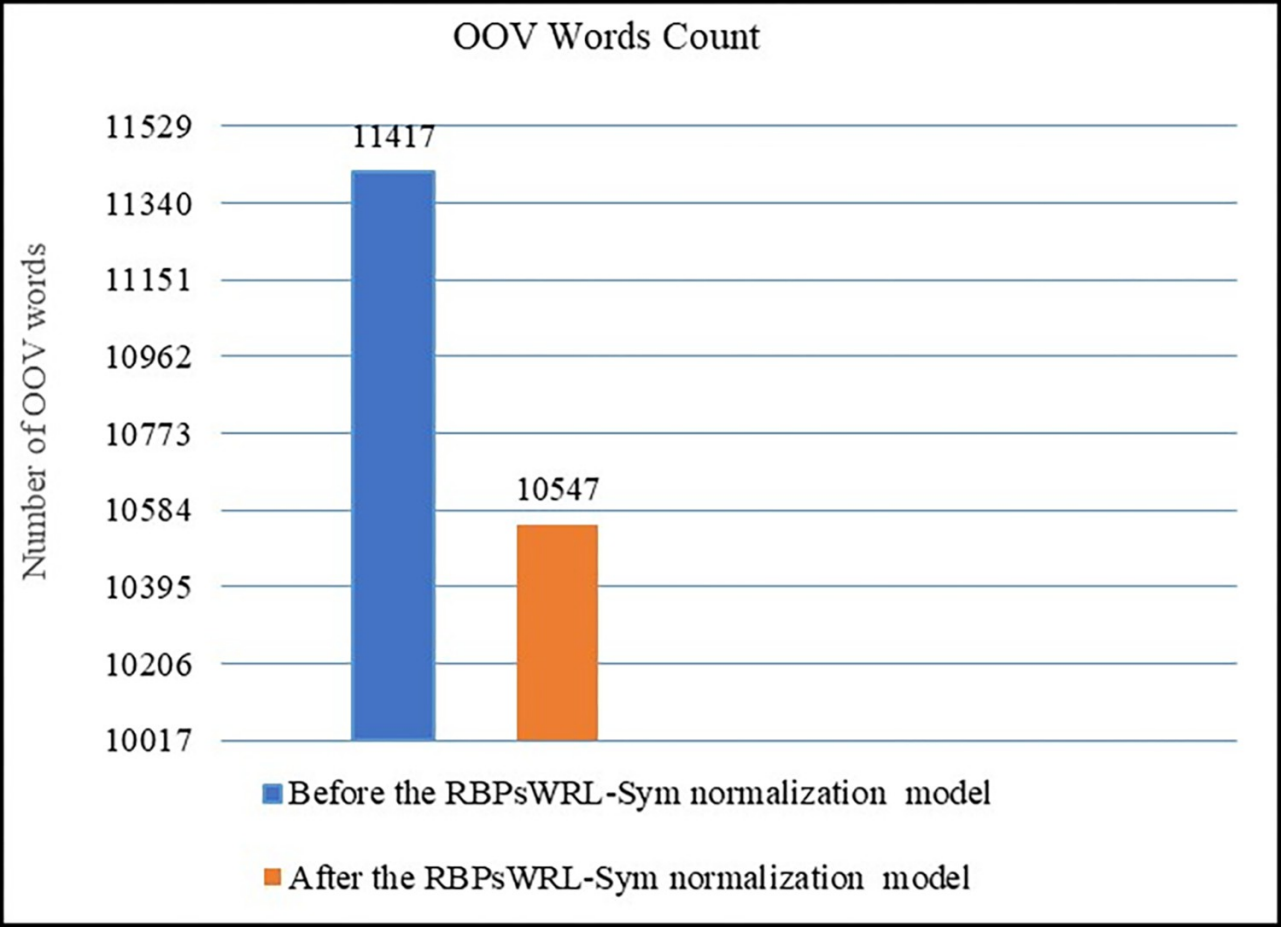
The number of OOV words in the Waseem and Hovy dataset [51] before and after the RBPsWRL-Sym model had been applied.

The first experiment was conducted to determine the percentage by which the proposed normalization model (RBPsWRL Sym) was able to decrease the level of OOV words in the dataset by [51]. The dataset by [51] was first put through the Pyspellchecker spelling checker by [56] to determine the number of known and unknown OOV words that it contained. After the RBPsWRL Sym model had been applied, the dataset was passed through the Pyspellchecker spelling checker again, as explained in Subsection 3.2.3 (a), to calculate the amount of known and unknown words remaining. Fig 19 depicts the amount of known and unknown OOV words in the dataset by [51] before and after the RBPsWRL Sym model has been applied. The RBPsWRL Sym model decreased the number of OOV words from 11417 to 10547, yielding an 8% reduction.

### 4.2 Experiment 2: Performance of the RBPsWRL-Sym normalization model on the Davidson et al. dataset [52]

The performance of the RBPsWRL-Sym was compared to that of other normalization methods using the hate speech dataset by [52]. Common metrics such as the F1-score, Precision, and recall [2,24,38,58] were taken to measure the robustness of the normalization model. Precision measures the variance among the quantity of correct words and incorrect words after a repeated letters normalization algorithm has been applied (Eq 1). In contrast, recall measures the difference between the number of correct words and non-normalized words (Eq 2). Non-normalized words are words that normalization models are unable to normalize. Precision and recall were evaluated at the word level, while the F1 score was used to assess the reliability of an experiment (Eq 3).


$$\mathrm{Precision}=\frac{Correct}{Correct+Incorrect}$$ (1)

$$\mathrm{Recall}=\frac{Correct}{Correct+non\_normalized}$$ (2)

$$F1\;Score=\frac{2*(Precision*Recall)}{Precision+Recall}$$ (3)

**Fig 20:**
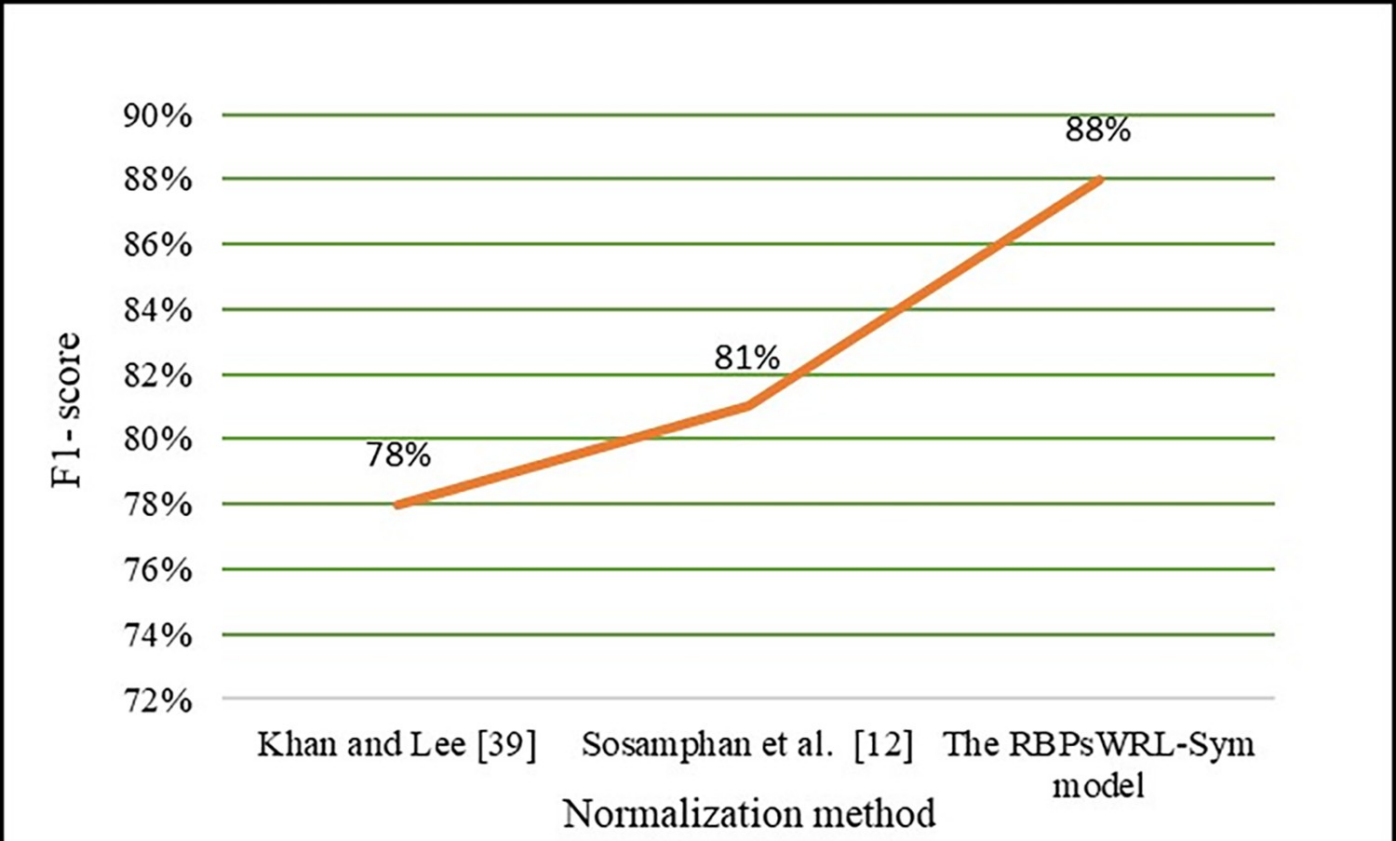
F1-scores of the RBPsWRL-Sym model vs two benchmark normalization models.

**Fig 21:**
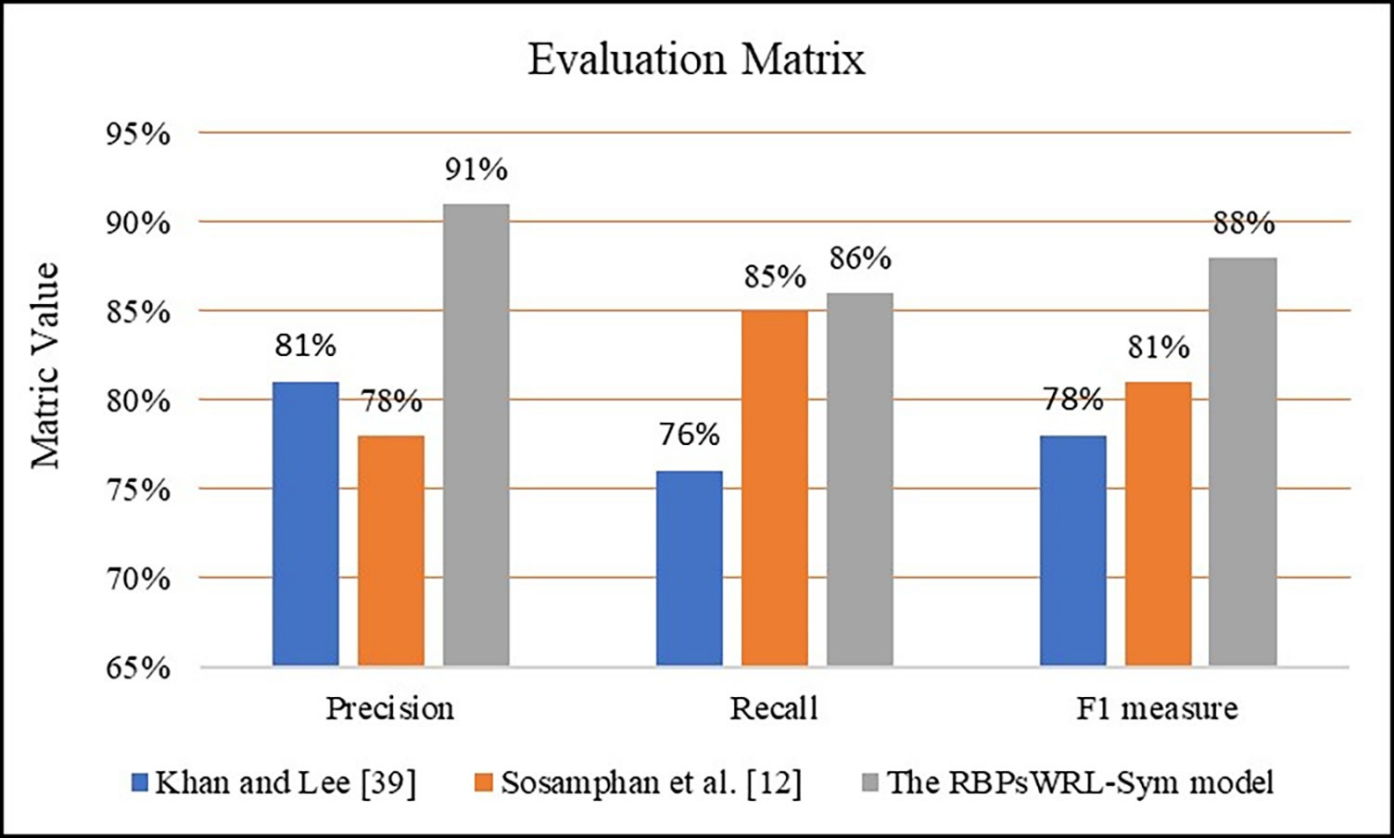
Precision, recall, and F1 score ratio of the RBPsWRL-Sym model vs. two benchmark normalization models.

For the evaluation, 300 words were automatically and randomly extracted from the dataset by [52] and put through the RBPsWRL-Sym model. Table 5 lists the number of correct, incorrect, and non-normalized words. These same 300 words were then put through the normalization methods proposed by [12,39]. Both these models were able to use the regular expression method and a spell-check algorithm to normalize OOV words with repeated letters resulting in impressive outcomes. Table 5 provides a comparison of the outcomes of the RBPsWRL- Sym model and that of the normalization models proposed by [12,39]. As seen in Fig 20, the RBPsWRL-Sym model increased the F1 score from 78% and 81% to 88%. Therefore, according to the F1 scores, the RBPsWRL-Sym model performed 9% better than the normalization model by [12] and 13% better than that of [39] (Figs 20 and 21). Both these methods truncate repeated letters followed by a spelling correction algorithm.

**Table 5:** Comparison of the performance of the RBPsWRL-Sym model and two extant normalization methods on the dataset by [52].

Normalization Methods	Word count		
	Correct words	Incorrect words	Non-normalized words
Sosamphan et al. [12]	206	58	35
Khan and Lee [39]	215	52	33
The RBPsWRL-Sym model	**239**	**23**	38

### 4.3 Experiment 3: Performance of the RBPsWRL-Sym normalization model on the hate speech detection performance

The final experiment aimed to assess the extent to which the proposed normalization model (RBPsWRL Sym) enhanced the performance of BiLSTM on the dataset by [51] in terms of percentage improvement. In other words, this evaluation aimed to ascertain that the performance of the BiLSTM classifier experiences enhancements due to the RBPsWRL Sym normalization model implementation.

The development of the BiLSTM model relied on several open-source Python libraries including NumPy, Pandas, Matplotlib, and Keras. These libraries were utilized to create the entire model. The Natural Language Toolkit (NLTK) Python library also plays a role in the proposed model. Furthermore, the scikit-learn library provides a comprehensive interface for supervised and unsupervised machine learning in Python.

The classifier will undergo two testing phases: first on the original dataset without any proposed normalization model, then independently after applying the normalization model. Keras offers essential text data preprocessing library utilities within deep learning. Among these, the text_to_word_sequence() function stands out as it breaks down text into a collection of individual words.

The dataset will first be accessed using the Pandas library. It will be partitioned into a training set comprising 80% of the data and a test set containing the remaining 20%. The next step entails preprocessing the input texts, encompassing various user posts and their associated classes. Keras includes the Tokenizer class, designed to compile textual data for deep learning purposes. To use the Tokenizer effectively, it should be instantiated and configured to work with numeric or raw text documents. The Adam optimizer, a gradient-based optimization technique for stochastic objective functions, within the Keras framework was employed. Hyperparameter tuning plays a crucial role in deep learning algorithms, including adjustments to factors like the optimization method, as highlighted by [59]. Table 6 offers the parameters used for training the BiLSTM based on the previous hate speech methods such as the experiments by [60,61] and the practical issue.

**Table 6:** BiLSTM parameter setting.

Parameter	Value
Vocabulary Size	5000
Embedding dimension	100
Epoch	50
Batch Size	64
Learning rate	0.001
Input Length	200
LSTM neurouns	200
Dropout rate	0.5

The Sparse_categorical_crossentropy was used for loss and softmax activation function to generate a probability as the resulting output. The fit method (model, fit()) in Keras trained the model for a certain amount of epochs. To terminate the training, the Keras callback function was used, initially denoted as EarlyStopping, while configuring specific parameters. TensorFlow 2.0 and Keras were used in Python 3.7.4 to create the BiLSTM neural framework. Workstation Intel (R) Core (TM) i7-9750H CPU was used running at 2.60GHz and 2.59GHz with Windows 10. Different metrics, including precision, recall, and the F1 score, were employed to gain insights into the efficiency of the model in terms of its ability to classify instances accurately. Table 7 presents the output results for the BiLSTM classifier before and after using the proposed normalization model. Table 7 demonstrates that all the metrics in all the classes have significantly improved after using the proposed RBPsWRL Sym normalization model.

**Table 7:** The performance of the BLSTM before and after the RBPsWRL Sym normalization model.

Category	Metric	BiLSTM before using the RBPsWRL Sym model	BiLSTM after applying the RBPsWRL Sym normalization model
Racism	Precision	0.49	**0.83**
	Recall	0.25	**0.60**
	F1-score	0.33	**0.70**
Sexism	Precision	0.48	**0.75**
	Recall	0.16	**0.72**
	F1-score	0.24	**0.74**
None	Precision	0.73	**0.86**
	Recall	0.91	**0.93**
	F1-score	0.81	**0.89**
Macro average over all categories	Precision	0.57	**0.82**
	Recall	0.44	**0.75**
	F1-score	0.46	**0.78**

Table 7 clearly shows the positive impact of applying the proposed method to the hate speech detection model. Results of the experiment demonstrated a considerable improvement in all measures across all classes when the suggested RBPsWRL Sym normalization model was used.

## 5. Discussion

[44] specified very few rules for normalizing Greek text; specifically, repeated letters at the beginning or end of a word should be truncated to one, while those in the middle should be truncated to two. However, in the case of English texts, different handling conditions are required to handle letters that are repeated at different positions in a word and when multiple letters are repeated in the same word. The proposed RBPsWRL-Sym normalization model was based on the concept of using the regular expression method to remove repeated letters. However, rather than using the method of the regular expression, three separate algorithms, namely: TRL1, TRL2, and TRLM2 were developed and used in the proposed RBPsWRL-Sym normalized model to perform the same function and manipulate repeated letters at any position within a word.

The performance evaluation indicated that the RBPsWRL-Sym model outperformed two benchmark models (Experiment 2 section, Table 6). As seen in Fig 21, the precision (91%), recall (86%), and F1-score (88%) of the RBPsWRL-Sym model are the highest of the three evaluated models namely [12,39]. The RBPsWRL-Sym model performed the best as it uses an algorithm that relies on the position of letter repetitions in a word and the use of rule-based repeated letter patterns. Therefore, normalization methods that are designed based on repeated letter positions are more effective.

The RBPsWRL-Sym model was also independent of constructing a specific dictionary or providing labeled data. The RBPsWRL-Sym model decreased the size of OOV words in the dataset by [51] by 8%. Although an 8% reduction may not seem impressive, it is noteworthy that a majority of the remaining OOV words are not words with repeated letters, but are rather slangs, abbreviations, and emoticons.

There are some OOV words with repeated letters that the RBPsWRL-Sym model could not entirely correct, such as ‘lllllooool’, ‘fucccccc’, and ‘awwwww’. Although the RBPsWRL-Sym model correctly removed the repeated letters of these words: [LOL, FUC, AW], these words could not be found by searching the SymSpell dictionary. These words demand a different handling for further work since they contain slang expressions, unconventional language, or abbreviated terms.

Additional experiments were conducted to examine the impact of the RBPsWRL Sym normalization model on the detection performance using BiLSTM on the dataset by [51]. The experiment outcomes showed that by employing the suggested RBPsWRL Sym normalization model, all the metrics across all the classes showed significant improvement.

The manual examination of the unknown words revealed that additional effort was needed because various letters appeared in varied locations from word to word. Consequently, creating an algorithm around these patterns led to better normalization outcomes. However, this might vary from language to language, resulting in various rules dependent on the pattern observed. Even though there have been numerous studies on the topic, several challenges persist across most solutions [62].

## 6. Contributions

In this section, we outline the primary contributions of this work and highlight the key distinctions from existing methods.

New Algorithm: We introduced a new algorithm for normalizing words with repeated letters that significantly advance the state-of-the-art. Our model is based on multiple rules regarding the position of repeated letters in a word and the SymSpell spelling correction algorithm.

Improved Performance: We demonstrated that our model has superior performance compared to existing solutions. In particular, we reported an 8% reduction in OOV words in hate speech tweets. Likewise, 9% and 13% of the improvements are higher than that of the models proposed by two extant studies, highlighting the advantages of our model.

Efficiency: The new normalization model is an unsupervised method that does not require a special dictionary or annotated data. This is achieved by combining rule-based patterns of words with repeated letters and the SymSpell spelling correction algorithm.

Robustness: We addressed the issue of words with repeated letters, which has been challenging in this domain. Our model can determine the correctness of the in-vocabulary (IV) replacement words under various conditions.

Scalability: Our model is designed to improve the detection performance of hate speech. We showed its effectiveness in the detection performance which demonstrated a significant improvement after normalization.

Ultimately, we offer a new method to normalize words with repeated letters and swap them out for their lexical equivalents. We also demonstrated that our approach outperforms earlier approaches and produces better outcomes. Through our significant contributions, we drive the field of hate speech detection to new heights by overcoming technical obstacles. Our development of innovative algorithms addresses existing challenges and sets the stage for the emergence of more dependable and inclusive hate speech detection systems within the digital landscape.

## 7. Conclusion and future work

This study proposed a new normalization method, RBPsWRL-Sym. The proposed RBPsWRL-Sym normalization model incorporates a rule-based pattern of letters repeated in words and spelling correction using the SymSpell algorithm. It was trained using a hate speech dataset, which will later be used in the pre-processing stage of an enhanced hate speech detection pipeline model. The proposed model was built by analyzing the position of letter repetitions within words and then developing several algorithms to match these different patterns. Therefore, the RBPsWRL-Sym model could find the most appropriate replacement IV for OOV words with repeated letters. Three different evaluation experiments were conducted to examine the effectiveness of the proposed RBPsWRL Sym normalization model. The first internal evaluation involves counting the OOV words from the dataset by [51] and tracking the reduction amount. The second external evaluation involves a different hate speech dataset than the training dataset, which was used to evaluate the performance of the RBPsWRL-Sym against that of other normalization techniques. The final assessment explores the impact of the REPsWRL-Sym normalization model on the effectiveness of hate speech identification.

Internal and extrinsic evaluations of the proposed model indicated that it could decrease the number of OOV words in hate speech tweets by 8%. As indicated by its F1 score, the RBsPWRL-Sym model was also 9% and 13% more adept at normalizing words with repeated letters than the two benchmarks. In addition, the RBPsWRL-Sym normalization model was tested on the extent of its impact on the performance of the detection model by BiLSTM. The findings of the experiment demonstrated a beneficial impact on the BiLSTM hate speech detection model by using the proposed RBPsWRL Sym normalization model.

Therefore, rule-based patterns and spelling correction algorithms can be combined to develop efficient text normalization models for words with repeated letters in hate speech. Several other categories of OOV words, such as slang, emoticons, and abbreviations, can be found in social media messages like tweets. However, the number of these OOV words should be reduced for better feature representation. Therefore, the authors intend to: (1) extend the capabilities of the proposed RBPsWRL-Sym normalization model to handle one or more of these forms of OOV words and (2) investigate the efficacy of the proposed method in other detection methods or other types of datasets
